# Genome-wide association and genomic prediction of resistance to viral nervous necrosis in European sea bass (*Dicentrarchus labrax*) using RAD sequencing

**DOI:** 10.1186/s12711-018-0401-2

**Published:** 2018-06-08

**Authors:** Christos Palaiokostas, Sophie Cariou, Anastasia Bestin, Jean-Sebastien Bruant, Pierrick Haffray, Thierry Morin, Joëlle Cabon, François Allal, Marc Vandeputte, Ross D. Houston

**Affiliations:** 1 The Roslin Institute¸Royal (Dick) School of Veterinary Studies, University of Edinburgh, Easter Bush, Midlothian, EH25 9RG Scotland, UK; 2 Ferme Marine De Douhet, BP 4, 17840 La Brée Les Bains, France; 3 SYSAAF, LPGP-INRA, Campus de Beaulieu, 35042 Rennes Cedex, France; 4 French Agency for Food, Environmental and Occupational Health and Safety (ANSES), Ploufragan-Plouzané Laboratory, Viral Fish Pathology Unit, National Reference Laboratory for Regulated Fish Diseases, Bretagne Loire University, Technopôle Brest-Iroise, BP 70, 29280 Plouzané, France; 5 MARBEC, Université de Montpellier, Ifremer-CNRS-IRD-UM, Palavas-les-Flots, France; 6 GABI, INRA, AgroParisTech, Université Paris-Saclay, 78350 Jouy-en-Josas, France

## Abstract

**Background:**

European sea bass (*Dicentrarchus labrax*) is one of the most important species for European aquaculture. Viral nervous necrosis (VNN), commonly caused by the redspotted grouper nervous necrosis virus (RGNNV), can result in high levels of morbidity and mortality, mainly during the larval and juvenile stages of cultured sea bass. In the absence of efficient therapeutic treatments, selective breeding for host resistance offers a promising strategy to control this disease. Our study aimed at investigating genetic resistance to VNN and genomic-based approaches to improve disease resistance by selective breeding. A population of 1538 sea bass juveniles from a factorial cross between 48 sires and 17 dams was challenged with RGNNV with mortalities and survivors being recorded and sampled for genotyping by the RAD sequencing approach.

**Results:**

We used genome-wide genotype data from 9195 single nucleotide polymorphisms (SNPs) for downstream analysis. Estimates of heritability of survival on the underlying scale for the pedigree and genomic relationship matrices were 0.27 (HPD interval 95%: 0.14-0.40) and 0.43 (0.29–0.57), respectively. Classical genome-wide association analysis detected genome-wide significant quantitative trait loci (QTL) for resistance to VNN on chromosomes (unassigned scaffolds in the case of ‘chromosome’ 25) 3, 20 and 25 (*P* < 1e06). Weighted genomic best linear unbiased predictor provided additional support for the QTL on chromosome 3 and suggested that it explained 4% of the additive genetic variation. Genomic prediction approaches were tested to investigate the potential of using genome-wide SNP data to estimate breeding values for resistance to VNN and showed that genomic prediction resulted in a 13% increase in successful classification of resistant and susceptible animals compared to pedigree-based methods, with Bayes A and Bayes B giving the highest predictive ability.

**Conclusions:**

Genome-wide significant QTL were identified but each with relatively small effects on the trait. Tests of genomic prediction suggested that incorporating genome-wide SNP data is likely to result in higher accuracy of estimated breeding values for resistance to VNN. RAD sequencing is an effective method for generating such genome-wide SNPs, and our findings highlight the potential of genomic selection to breed farmed European sea bass with improved resistance to VNN.

**Electronic supplementary material:**

The online version of this article (10.1186/s12711-018-0401-2) contains supplementary material, which is available to authorized users.

## Background

European sea bass (*Dicentrarchus labrax*) is a popular and valuable species for Mediterranean aquaculture, with a production volume of more than 150,000 tons [1]. Viral nervous necrosis (VNN) is a commonly-encountered pathogen, which has been detected in more than 70 wild or cultured marine and fresh water species [2]. Frequent mass mortalities of sea bass due to VNN have been reported, especially during the summer period, with fish being particularly susceptible during the larval and juvenile stages [3]. The disease is considered to be a primary problem in Mediterranean mariculture [4] and, to date, there is no fully effective therapeutic agent or vaccine to tackle it [5]. Selective breeding can be a valuable tool to prevent the detrimental effects of disease outbreaks in farmed livestock and fish [6]. Moderate to high heritabilities for disease resistance have been reported in numerous aquaculture species, which indicates that genetic progress is possible through selective breeding [7]. For resistance to VNN in sea bass, a moderate heritability of 0.26 was recently reported [8], which implies that selective breeding has potential as a component of VNN prevention and control.

Recent technological advances in genome-wide sequencing and genotyping technology have facilitated cost-effective generation of genome-wide marker data, even in non-model organisms [9]. Such genotyping data can be used in selective breeding programs to increase the accuracy of breeding value predictions for selection candidates, as compared to the classical breeding approach where breeding values are typically estimated at the family level using pedigree data [10, 11]. The genetic architecture of most traits relevant to farmed animal production (e.g. growth and disease resistance) is polygenic [12, 13], which explains why the application of marker-assisted selection in aquaculture has had limited success, with a few exceptions associated with major-effect loci [14–17]. Therefore, as for terrestrial livestock, genomic prediction [18] is an effective approach to improve selection accuracy compared to traditional pedigree-based approaches in aquaculture breeding programs [19–21].

Restriction-site associated DNA (RAD) sequencing is a reduced representation high-throughput sequencing technique for the concurrent detection and genotyping of single nucleotide polymorphisms (SNPs) in multiplexed samples, each containing a unique nucleotide barcode [22]. RAD sequencing and similar genotyping-by-sequencing techniques rely on the digestion of genomic DNA with a restriction enzyme and subsequent high-depth sequencing of the flanking regions. Such genotyping-by-sequencing techniques have been applied in a wide range of aquaculture species [23], both for genome-wide association studies (GWAS) [24–26] and genomic selection studies [27–31].

The aim of this study was to investigate genetic resistance to VNN in sea bass juveniles, using a RAD sequencing approach to generate genome-wide SNP data from 1538 individual disease-challenged fish. Heritability estimates were obtained using both pedigree and genomic relationship matrices for survival status (dead/alive) on the underlying liability scale. GWA approaches were used to test associations of both individual SNPs and genomic regions with resistance to VNN. Finally, genomic prediction of breeding values for resistance to VNN was tested using several approaches to evaluate the potential of genomic selection for genetic improvement of resistance to VNN in seabass.

## Methods

### Sample collection and disease challenge

The population of fish for the VNN disease challenge was obtained by artificial fertilization of 18 dams and 49 sires from the Ferme Marine de Douhet breeding nucleus (Oléron, France). Five factorial mating blocks, each comprising 9 to 10 sires and 3 to 5 dams, were created in February 2014. The fertilized eggs of each dam were transferred to individual incubators. Following egg hatching, equal numbers of larvae from each incubator were transferred in a single tank and reared in common environmental conditions.

The fish (at a size of ~ 10 g) were challenged at the Unité de Pathologies Virales des Poissons—ANSES facility (Plouzané, France). Autopsies and extensive bacteriological and virological analyses were carried out on receipt of fish to confirm their health status. After 4 weeks of acclimatization, 1990 individuals distributed in three tanks were immersed for 2 to 3 h in static seawater, containing 1 × 10^5^ 50% tissue culture infective dose (TCID_50_) per ml of the W80 betanodavirus strain, which was produced and titered in striped snakehead cells (SSN-1), as previously described [32]. Strain W80 belongs to the redspotted grouper nervous necrosis virus (RGNNV) genotype, the most common NNV type in the Mediterranean area [33]. A negative control tank that consisted of sea bass juveniles from the same population (n = 200) immersed with non-infected SSN-1-cell supernatant was also included. After infection, all fish were maintained at 27 ± 1 °C in an open water circuit. A pre-test of the experimental conditions was conducted to confirm the virulence of the pathogen.

### RAD library preparation and sequencing

DNA was extracted from fin samples of the challenged fish using the REALPure genomic DNA extraction kit (Durviz S.L.) and treated with RNase. Each sample was quantified by spectrophotometry (Nanodrop), quality-assessed by agarose gel electrophoresis, and finally diluted to a concentration of 20 ng/µL in 5 mmol/L Tris, pH 8.5 using a Qubit Fluorometer (Invitrogen).

The protocol for RAD library preparation followed the methodology originally described in Baird et al. [22]. Briefly, each sample (0.72 µg parental DNA per 0.24 µg offspring DNA) was digested at 37 °C for 60 min with the high-fidelity restriction enzyme *Sbf*I (that recognises the CCTGCA|GG motif) − (New England Biolabs; NEB) using 6 U *Sbf* I per µg genomic DNA in 1 × Reaction Buffer 4 (NEB) at a final concentration of 1 µg DNA per 50 µL reaction volume. Reactions (12 µL final volumes) were then heat-inactivated at 65 °C for 20 min. Individual specific P1 adapters, each with a unique 5–7 bp barcode, were ligated to the *Sbf* I-digested DNA at 20 °C for 60 min by adding 1.8/0.6 µL of 100 nmol/L P1 adapter, 0.45/0.15 µL of 100 mmol/L rATP (Promega), 0.75/0.25 µL 10 × reaction buffer 2 (NEB), 0.36/0.12 µL T4 ligase (NEB, 2 M U/mL) and reaction volumes for each parental/offspring sample were completed to 45/15 µL with nuclease-free water. Following heat inactivation at 65 °C for 20 min, ligation reactions were slowly cooled down to room temperature (over 1 h), then combined in appropriate multiplex pools. Shearing (Covaris S2 sonication) and initial size selection (300 to 600 bp) by agarose gel separation was followed by gel purification, end repair, dA overhang addition, P2 (individual specific adapters) paired-end adapter ligation, library amplification, as in the original RAD protocol [22, 34]. A volume of 150 µL of each amplified library (16 to 18 PCR cycles, library-dependent) was size-selected (400 to 700 bp) by gel electrophoresis [35]. Following a final gel elution step into 20 µL EB buffer (MinElute Gel Purification Kit, Qiagen), 66 libraries (24 animals each) were sent to BMR in Italy, for sequencing. Libraries were run in 14 lanes of an Illumina NextSeq 500, using 75 base paired-end reads (v2 chemistry). The sequence reads were deposited at the NCBI Sequence Read Archive (SRA) under the accession number PRJNA407892.

### SNP identification and genotyping

Sequence reads of low quality, with a missing restriction site, or with ambiguous barcodes and PCR duplicates were discarded. The remaining reads were aligned to the European sea bass reference genome [36] assembly *GCA_000689215.1* using the Bowtie2 program [37]. Aligned reads were sorted into RAD loci and genotypes were called using Stacks software 1.4 [38]. The likelihood-based SNP calling algorithm [39] implemented in Stacks evaluates each nucleotide position at each RAD-locus of all individuals, using a maximum likelihood approach to differentiate true SNPs from putative sequencing errors. SNP data for downstream analyses were obtained after the following quality control (QC) steps: RAD loci were formed using a minimum stack depth of 10 (parental samples) or 5 (offspring samples) reads. Only RAD loci with a maximum of two SNPs were considered for downstream analysis. SNPs that had a minor allele frequency (MAF) less than 0.05 and more than 25% missing data and those that deviated from expected Hardy–Weinberg equilibrium (*P* < 1e–06; parental samples) were discarded.

### Parentage assignment

Parentage assignment was performed with the R/hsphase program [40] using all SNPs that passed QC and allowing for a maximum genotyping error of 3.5%. The pedigree obtained using this approach was further validated for potential erroneous assignments using the FImpute software [41].

### Estimation of heritabilities

Variance components were estimated using the R/BGLR software [42]. The probit link function was used to connect the observed binary phenotype (0 = dead, 1 = alive) with the latent variable (the underlying liability). The following model was applied:

1 $${\mathbf{l}} = {\mathbf{Xb}} + {\mathbf{Zu}} + {\mathbf{e}} ,$$

where ${\mathbf{l}}$ is a vector of latent variables, ${\mathbf{b}}$ a vector of the fixed effects (intercept and tank), ${\mathbf{X}}$ the incidence matrix relating phenotypes with the fixed effects, ${\mathbf{Z}}$ the incidence matrix relating phenotypes with the random animal genetic effects, ${\mathbf{u}}$ the vector of random animal genetic effects with the following distribution $N\left( {0,{\mathbf{A}}\upsigma_{\text{g}}^{2} } \right)$, where ${\mathbf{A}}$ is the pedigree-based relationship matrix, which was replaced with the genomic relationship matrix ${\mathbf{G}}$ [43] for certain analyses as described below, $\upsigma_{\text{g}}^{2}$ is the additive genetic variance, and ${\mathbf{e}}$ is a vector of residuals with the following distribution $N\left( {0,{\mathbf{I}}\upsigma_{\text{e}}^{2} } \right)$, where $\upsigma_{\text{e}}^{2}$ is the residual variance and **I** the identity matrix.

The parameters of this model were estimated by Monte Carlo Markov chain (MCMC) Gibbs sampling (11 million iterations; burn-in: 1 million; thin: 1000). Convergence of the resulting posterior distributions was assessed both visually (inspecting the resulting MCMC plots) and analytically using the R/coda v0.19-1 software [44]. Heritability for survival on the underlying scale was estimated as:

$$h^{2} = \frac{{\upsigma_{\text{g}}^{2} }}{{\upsigma_{\text{g}}^{2} +\upsigma_{\text{e}}^{2} }} ,$$

where $\upsigma_{\text{g}}^{2}$ is the estimate of the additive genetic variance and $\upsigma_{\text{e}}^{2}$ the residual variance, which was set equal to 1 because it is not identifiable in threshold models [45, 46].

### Genome-wide association study (GWAS)

To test the association of individual SNPs with resistance to VNN, a ‘classical’ genome-wide association study (CGWAS) was performed using the R/gaston software [47]. The mixed model applied for overall survival on the observed scale had the same format as in Eq. (1) but with the addition of the genotype of an individual SNP as a fixed effect. Variance components were estimated using the penalized quasi-likelihood approach [48]. The genome-wide significance threshold was calculated using a Bonferroni correction (0.05/N), where N represents the number of QC-filtered SNPs across the genome.

In addition to the CGWAS approach, weighted genomic best linear unbiased predictor (WGBLUP) was performed [49] to estimate SNP effects by using genomic estimated breeding values (GEBV) [50]. SNP weights were estimated using non-overlapping windows of 10 adjacent SNPs. Explained additive genetic variance was estimated using subsequent non-overlapping windows including adjacent SNPs within a distance of 0.5 Mb. Initially, the weighted genomic relationship matrix was created following the method of VanRaden [43] as:

$${\mathbf{G}}^{*} = {\mathbf{ZDZ}}^{\prime }{\mathbf{q}},$$

where ${\mathbf{Z}}$ is the design matrix relating genotypes of each SNP with phenotypes, ${\mathbf{D}}$ is a weight matrix for all SNPs, and ${\mathbf{q}}$ is a weighting vector derived from the observed SNP frequencies. Briefly, WGBLUP was carried out as follows [49]:

- Initialize ${\mathbf{D}} = {\mathbf{I}}$ and ${\text{t}} = 1$, where ${\mathbf{I}}$ is the identity matrix and ${\text{t}}$ is the iteration number;
- Calculate $${\mathbf{G}}^{*}$$;
- Estimate GEBV using GBLUP;
- Estimate the SNP effects from GEBV based on the equation ${\hat{\varvec{\alpha }}} = {\mathbf{qDZ}}^{\prime }{\mathbf{G}}^{ *} {\hat{\mathbf{u}}}$, where ${\hat{\varvec{\alpha }}}$ represents the vector of SNP effects and ${\hat{\mathbf{u}}}$ vector of GEBV;
- Calculate the weight for matrix ${\mathbf{D}}$ for each SNP: $d_{ii}^{{\left( {t + 1} \right)}} = \frac{{\mathop \sum \nolimits_{i = 1}^{n} u_{i}^{2} }}{n}$, where $n = 10$ represents number of adjacent SNPs;
- Normalize the weights of SNPs such that the total genetic variance remains constant;
- Loop to step (d) (three iterations).

The percentage of additive genetic variance explained by each genomic region was estimated by non-overlapping windows of 0.5 Mb as follows:

$$\frac{{{\text{Var}}\left( {{\varvec{\upalpha}}_{\text{i }} } \right)}}{{\upsigma_{\alpha }^{2} }} \times 100\% = \frac{{{\text{Var}}\left( {\mathop \sum \nolimits_{\text{i}}^{{{\text{i}} = {\text{w}}}} {\text{z}}_{\text{i }} \widehat{{{\text{u}}_{\text{i }} }}} \right)}}{{\upsigma_{\alpha }^{2} }} \times 100\% ,$$

where ${\text{w}}$ denotes group of SNPs within the tested window.

This analysis was performed using the THRGIBBS1F90 program from the BLUPF90 software suite [51, 52] to estimate the GEBV, combined with three iterations using PreGSF90 and PostGSF90 [53].

### Genomic prediction

To assess the potential of genomic selection for improved resistance to VNN in sea bass breeding programs, the accuracy of GEBV was assessed and compared to EBV obtained with pedigree-based approaches. Missing genotypes for the SNPs that passed QC filters were imputed using FImpute [41]. Several commonly used genomic selection models were applied to the data using the R/BGLR software [42]: rrBLUP, BayesA, BayesB [18] and BayesC [54]. In addition, pedigree-based BLUP [10] was evaluated using the same software. The general form of the fitted models was as in Eq. (1):

2 $${\mathbf{l}} = {\mathbf{Xb}} + {\mathbf{Z}}{{\varvec{\upalpha}}} + {\mathbf{e}} ,$$

where ${\mathbf{Z}}$ is the incidence matrix relating the underlying liability with the genotypes and ${\varvec{\upalpha}}$ the vector of SNP effects using the corresponding prior distribution for each of the above Bayesian models. The parameters of each model were estimated by MCMC using Gibbs sampling (1.1 million iterations; burn-in: 100,000; thin: 100). Convergence of the resulting posterior distributions was assessed both visually (inspecting the resulting MCMC plots) and analytically using the R/coda v0.19-1 software [44].

Six-fold cross-validation was performed in order to test prediction accuracy of correctly classifying animals in the validation set as resistant or susceptible. The dataset was randomly split into sequential training (n = 1090 individuals) and validation sets (n = 218). The number of survivors and mortalities in each validation set was proportional to the overall survival of the challenged population. In the validation sets, the phenotypes of the animals were masked, and their (genomic) estimated breeding values—(G)EBV—were estimated based on the prediction model derived from the training set. This cross-validation procedure was repeated five times. Receiver operator characteristic (ROC) curves were used to assess the efficacy of classifying the animals as survivors or mortalities, using either the pedigree- or the genomic-based models. The area under the curve (AUC) metric [55, 56] was used to interpret the performance of the genomic prediction models, with values of 1 representing the perfect classifier.

## Results

### Disease challenge

The challenge study was conducted for 7 weeks, with mortalities recorded daily. Characteristic clinical signs of VNN were observed in each disease-challenged tank. In this experiment, the total percentage of mortality reached 48% (ranging from 40 to 56% depending on the tank), with four successive peaks of mortality (days 8, 15, 20 and 27); (see Additional file 1: Table S1). Presence of the virus was confirmed in the anterior kidneys and spleens of dead individuals at 1 week post-infection, using primary cell culture coupled with an indirect fluorescent antibody test (IFAT). No mortality was observed in the control tank. Two of the challenge tanks experienced a drop in oxygen concentration at day 27 post-infection, but the fast return to a normal situation within 24 to 48 h after the incident without observing abnormal behavior or excess mortality led to the conclusion that this temporary oxygen drop did not affect the rest of the experiment. Furthermore, oxygen-related mortalities (day 27 post-infection) were excluded, resulting in 1538 VNN-challenged animals used for downstream analysis.

### SNP identification and genotyping

In total, 6.34 million (s.d. = 3.95 million) and 2.83 million (s.d. = 1.38 million) sequence reads passed the QC filters for the parental and offspring samples, respectively. The mean number of putative RAD loci identified was 45,631 (s.d. = 7099) and 43,538 (s.d. = 4543) for parents and offspring, with mean coverages of 68× (s.d. = 32) and 26× (s.d. = 12), respectively. The RAD loci were distributed relatively evenly across the sea bass genome assembly (see Additional file 2: Table S2). Only animals with fewer than 30% missing genotype data were retained for downstream analysis (67 parents and 1322 offspring). A total of 17,004 putative SNPs were identified, of which 9195 passed the QC filters and were retained for downstream analysis (Table 1) and (see Additional file 3: Table S3). Animals (n = 11) with a genotypic similarity greater than 90% were discarded as potential replicated samples.

**Table 1:** Number of QC-filtered SNPs per chromosome

Chromosome	Corresponding scaffold (seabass_v1.0)	Number of markers
1	HG916827.1	330
2	HG916828.1	393
3	HG916829.1	321
4	HG916830.1	387
5	HG916831.1	367
6	HG916832.1	355
7	HG916833.1	293
8	HG916834.1	375
9	HG916835.1	237
10	HG916836.1	340
11	HG916837.1	370
12	HG916838.1	268
13	HG916839.1	371
14	HG916840.1	367
15	HG916841.1	336
16	HG916842.1	254
17	HG916843.1	145
18	HG916844.1	420
19	HG916845.1	390
20	HG916846.1	404
21	HG916847.1	376
22	HG916848.1	334
23	HG916849.1	322
24	HG916850.1	259
25	HG916851.1	1181
Total		9195

### Parentage assignment

**Fig. 1:**
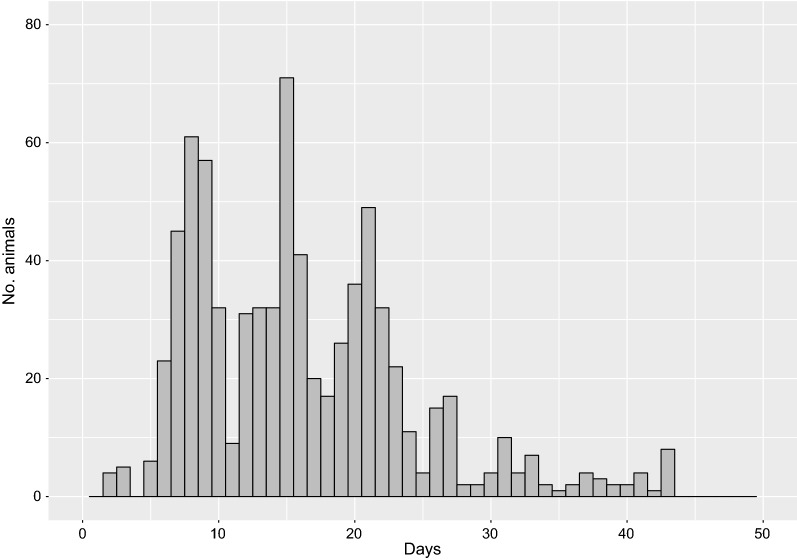
Daily mortality rates during the VNN disease challenge

Progeny were assigned to unique parental pairs, allowing for a maximum genotypic error of 3.5%. One thousand two hundred and ninety-seven offspring were uniquely assigned, which revealed that the challenged population (produced from the factorial batch-spawning design described above) comprised 140 full-sib families (48 sires and 17 dams), with 1 to 32 animals per family and a mean size of 9 (s.d. = 8). The contribution of individual dams to the population ranged from 1 to 267 animals, with a mean of 77 (s.d. = 68), while the contribution of sires ranged from 1 to 66, with a mean of 27 (s.d. = 18). Further testing of parentage assignment results using FImpute identified four animals as potentially mis-assigned. These individuals were discarded, which left 1293 animals originating from 48 sires and 17 dams in the final dataset used for the subsequent genetic analyses. The overall survival of these individuals in the VNN challenge was 42% (Fig. 1). The survival rate varied substantially among the offspring of individual sires (0–100%) and dams (0–69%), which indicates genetic variation for resistance to VNN.

### Heritability estimates

Estimates of heritability of overall survival on the underlying liability scale were significant and moderate, at 0.27 (highest posterior density, HPD 95% interval 0.14–0.40) using the pedigree relationship matrix and at 0.43 (HPD 95% interval: 0.29–0.57) using the genomic relationship matrix.

### Genome-wide association study (GWAS)

**Fig. 2:**
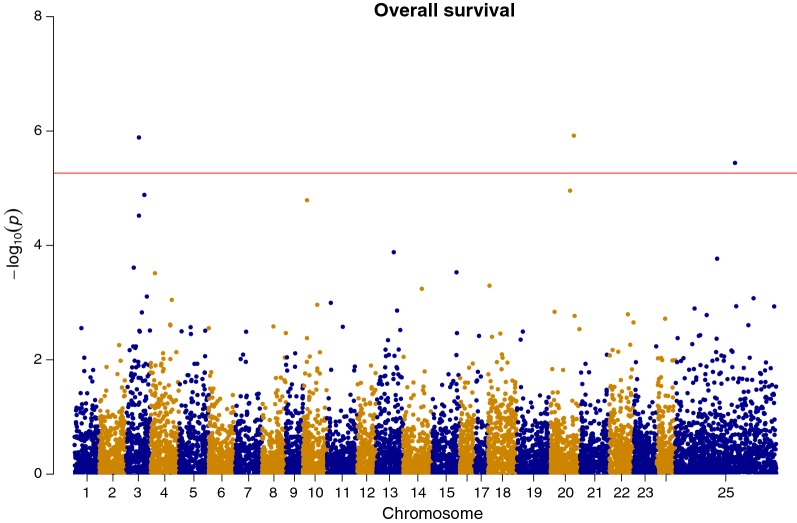
Genome-wide association plot for survival during the VNN challenge using single SNP GWAS

**Fig. 3:**
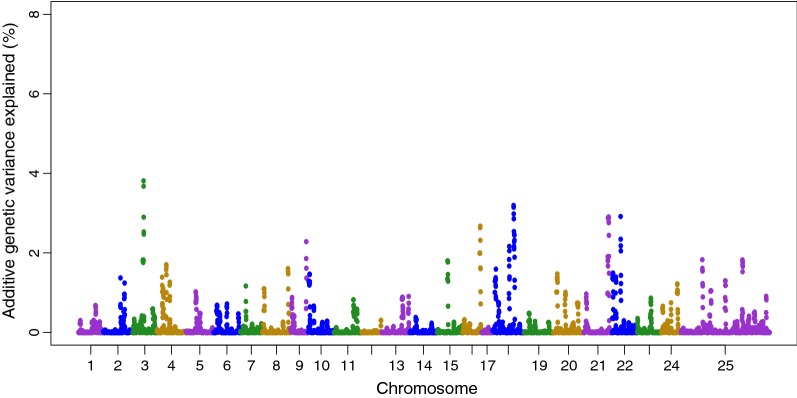
Genome-wide association plot for WGBLUP for survival during the VNN challenge, with the explained additive genetic variance calculated using non-overlapping windows of 0.5 Mb

In the CGWAS, SNPs that exceeded the genome-wide significance level were located on chromosomes 3, 20 and 25 (chromosome 25 corresponds to unassigned scaffolds and contigs of the reference genome assembly; *P* < 1e–05; R^2^: 0.03–0.05; Fig. 2). The WGBLUP analysis provided support for the putative QTL on chromosome 3, with the 0.5-Mb window containing the QTL-associated SNPs estimated to explain approximately 4% of the genetic variation. The putative QTL on chromosomes 20 and 25 were estimated to explain 1.5 and 2% of the genetic variation, respectively, in the WGLUP. Additional putative QTL regions that explained more than 2% of the additive genetic variance were identified on chromosomes 9, 18, 21 and 22 (Fig. 3).

### Genomic prediction

**Fig. 4:**
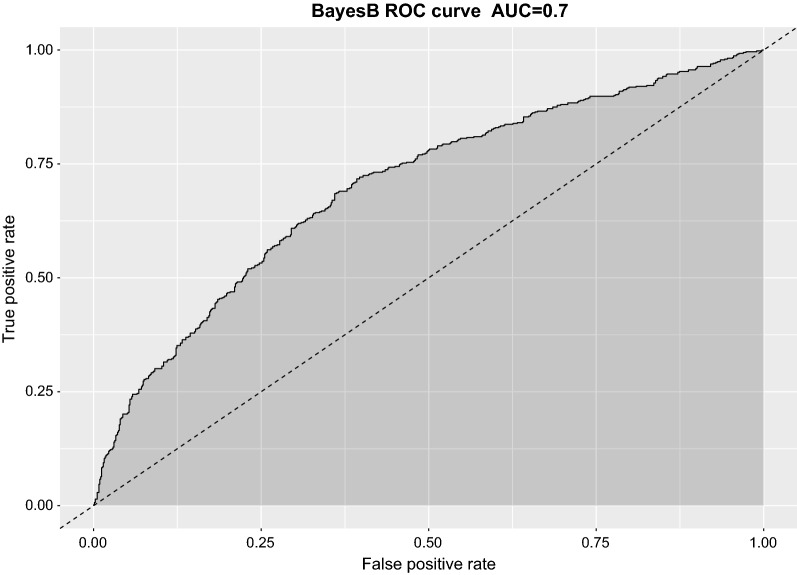
ROC curve and corresponding AUC metric for BayesB-based predictions of sea bass survival or mortality during the VNN challenge. The plot was obtained from aggregation of a sixfold cross validation scheme

The genomic prediction models successfully classified animals of the validation set with a success rate ranging from 67 to 70%, representing improvements of 8 to 13% over pedigree-based BLUP (PBLUP) (Table 2, Fig. 4). The best classifications were obtained with the Bayes A and Bayes B methods, corresponding to a success rate of 70% (Table 2) for correct classification (based on the AUC values of the ROC curves; Fig. 4).

**Table 2:** Percentage of VNN challenged sea bass with correctly predicted survival status for pedigree-based (PBLUP) and genomic prediction methods

Replication	PBLUP	rrBLUP	Bayes A	Bayes B	Bayes C
1^st^	0.63	0.67	0.70	0.71	0.67
2^nd^	0.62	0.66	0.69	0.70	0.67
3^rd^	0.62	0.67	0.70	0.69	0.68
4^th^	0.62	0.66	0.70	0.70	0.68
5^th^	0.62	0.67	0.70	0.70	0.69
Mean	0.62	0.67	0.70	0.70	0.68

Values obtained using area under curve (AUC) from ROC curves

Five replicates of sixfold cross-validation

## Discussion

European sea bass is a farmed species of paramount importance for Mediterranean aquaculture. Breeding for improved genetic resistance to VNN offers a promising avenue for reducing economic losses due to this disease and for improving animal welfare. A small number of family-based selective breeding programs exist for sea bass, with growth and disease resistance as the major target traits. Applying genomic information to selective breeding schemes facilitates direct selection for favorable alleles at major QTL (marker-assisted selection) and/or incorporation of genome-wide markers into the prediction of breeding values (genomic selection). Genomic prediction methods have been repeatedly shown to increase selection accuracy compared to pedigree-based approaches in aquaculture [29, 30, 57], which should translate to faster rates of genetic gain. High-density SNP arrays have been used for genomic selection, but these require substantial prior investment for development and application. As such, SNP arrays may be too expensive for routine genotyping in small to medium scale selective breeding programs, and more cost-effective genotyping methods are highly desirable. Techniques such as RAD sequencing, which require minimal prior investment, offer a cost effective alternative of generating genome-wide SNP datasets, even in the absence of *prior* genomic resources [22]. RAD sequencing and other genotyping-by-sequencing approaches are therefore becoming increasingly commonplace in aquaculture genetics and breeding [23].

Our study is the first application of high throughput sequencing to study genetic resistance of sea bass to VNN. The estimated heritability of resistance was moderate to high (ranging from 0.27 to 0.43). The heritability estimate obtained by using the pedigree relationship matrix (0.27) was in accordance with a recently estimated heritability (0.26) for resistance to VNN in several sea bass populations, also using pedigree [8]. These significant heritability estimates indicate that the aquaculture industry can benefit from the application of selective breeding for sea bass resistant to VNN. The reason that a higher heritability estimate was obtained when using the genomic relationship matrix (0.43) is not clear, but it is plausible that linkage disequilibrium generated by recent selective breeding may cause overestimation of additive genetic variance when using a genomic relationship matrix [58].

The CGWAS identified genome-wide significant QTL in several genomic regions (chromosomes 3, 20, 25, with chromosome 25 representing unassigned genome contigs and scaffolds). The single SNP CGWAS approach may lack statistical power to detect QTL when compared to methods in which all SNPs are used simultaneously, because of a false assumption of independence between SNPs [49]. WGBLUP is an approach that combines the computational efficiency of GBLUP with an increase in statistical power for QTL detection [50].

The genomic region that explained the highest percentage of additive genetic variance in the WGBLUP (~ 4%) was located on chromosome 3 and was also detected in the CGWAS. However, this may be an overestimation of the explained additive variance because threshold models often lead to overestimates of genetic variance [59]. In spite of some degree of discrepancy between the two GWAS approaches used, both highlight significant QTL that may play a role in host resistance to VNN, but also suggest that the trait is polygenic or ‘oligogenic’ in nature. In a similar study on resistance to VNN in Asian sea bass, no major QTL were identified [60], which supports the hypothesis that genetic resistance to VNN may be under the control of many genomic regions, each with a minor to moderate effect. In such situations, genomic selection is likely the most appropriate approach for using genetic markers to improve selective breeding.

The results from the genomic prediction approach used in the current study were encouraging for practical implementation of genomic selection for genetic resistance in sea bass. For traits like disease resistance, which cannot be measured directly on selection candidates, genomic selection benefits from the use of within-family genetic variation, compared to pedigree-based BLUP, which uses between-family genetic variation only [61]. Improvements in selection accuracies due to genomic prediction for disease resistance traits have been documented in various finfish aquaculture species, including Atlantic salmon [21], rainbow trout [29], and gilthead sea bream [30]. The AUC metrics derived from the ROC curves take the rate of both false positives and false negatives into consideration and have been routinely used to test the efficacy of prediction models for binary traits both in humans [56] and in livestock [62]. Interestingly, our study resulted in higher AUC values compared to livestock studies that used similar sample sizes and SNP density [62, 63], with the best performing models on average correctly classifying 70% of the validation set. This may be related to long-range linkage disequilibrium as a result of close genetic relationships between training and validation sets, which include large numbers of full and half siblings. Genetic relationships between reference and test populations are known to be a key factor that influences prediction accuracies in genomic selection [64]. However, reliance on these close genetic relationships for genomic prediction implies that generalization of these results to other populations and reference population designs should be undertaken with caution. Furthermore, additional testing of genomic prediction at varying SNP densities on a wider population is required to ascertain the appropriate SNP density for commercial application of genomic selection.

## Conclusions

We applied RAD sequencing to study the genetic basis of resistance to VNN in a large sample of juvenile sea bass derived from a commercial breeding program. RAD-derived SNPs allowed us to perform pedigree assignment in this batch-spawning species, estimate genetic parameters, perform GWAS, and test genomic selection. Genome-wide significant QTL were identified with minor to moderate effects on host resistance, on chromosomes 3, 20 and on an unassigned genome scaffolds. Genomic prediction using the RAD genotype data was effective, demonstrating significant improvement in prediction accuracy, and facilitating classification of surviving and mortality fish with a success rate of 70% using the cross-validation approach. This highlights the utility of genotyping-by-sequencing for genomic prediction of disease resistance in aquaculture species, and demonstrates that genomic selection can be an effective method for improving resistance to one of the most problematic infectious diseases in sea bass aquaculture.

## Additional files

**Additional file 1: Table S1.** VNN challenged sea bass. Phenotypic information of VNN challenged sea bass and pedigree structure.

**Additional file 2: Table S2.** Identified SNPs. Sequence information of identified SNPs.

**Additional file 3: Table S3.** SNP location. Location of SNPs on the sea bass reference genome v1_0.
